# Can Oxygen Set Thermal Limits in an Insect and Drive Gigantism?

**DOI:** 10.1371/journal.pone.0022610

**Published:** 2011-07-27

**Authors:** Wilco C. E. P. Verberk, David T. Bilton

**Affiliations:** Marine Biology and Ecology Research Centre, University of Plymouth, Plymouth, Devon, United Kingdom; Michigan State University, United States of America

## Abstract

**Background:**

Thermal limits may arise through a mismatch between oxygen supply and demand in a range of animal taxa. Whilst this oxygen limitation hypothesis is supported by data from a range of marine fish and invertebrates, its generality remains contentious. In particular, it is unclear whether oxygen limitation determines thermal extremes in tracheated arthropods, where oxygen limitation may be unlikely due to the efficiency and plasticity of tracheal systems in supplying oxygen directly to metabolically active tissues. Although terrestrial taxa with open tracheal systems may not be prone to oxygen limitation, species may be affected during other life-history stages, particularly if these rely on diffusion into closed tracheal systems. Furthermore, a central role for oxygen limitation in insects is envisaged within a parallel line of research focussing on insect gigantism in the late Palaeozoic.

**Methodology/Principal Findings:**

Here we examine thermal maxima in the aquatic life stages of an insect at normoxia, hypoxia (14 kPa) and hyperoxia (36 kPa). We demonstrate that upper thermal limits do indeed respond to external oxygen supply in the aquatic life stages of the stonefly *Dinocras cephalotes*, suggesting that the critical thermal limits of such aquatic larvae are set by oxygen limitation. This could result from impeded oxygen delivery, or limited oxygen regulatory capacity, both of which have implications for our understanding of the limits to insect body size and how these are influenced by atmospheric oxygen levels.

**Conclusions/Significance:**

These findings extend the generality of the hypothesis of oxygen limitation of thermal tolerance, suggest that oxygen constraints on body size may be stronger in aquatic environments, and that oxygen toxicity may have actively selected for gigantism in the aquatic stages of Carboniferous arthropods.

## Introduction

To predict species responses to global warming trends, it is paramount to understand the causal mechanisms underlying thermal limits. The idea of oxygen limitation as a mechanism setting upper thermal limits in animals was first expounded by Winterstein [1] and has since been greatly expanded by Pörtner and colleagues [2]–[5]. Recent work has extended this principle to lower thermal limits, and sees both upper and lower critical temperatures (*CT*_max_ and *CT*_min_) being coupled by the common mechanism of temperature-dependent oxygen limitation. As ectotherms warm the demand for oxygen in their tissues increases faster than the rate at which oxygen can be provided by cardiac and ventilatory processes, leading to a drop in whole-animal aerobic scope and a shift from aerobic to anaerobic metabolism. Similarly on cooling, metabolism slows, leading to insufficient ATP production in ventilatory muscles, reducing oxygen supply to tissues. Deleterious thermal effects are hypothesized to set in first at the whole-animal level, rather than lower hierarchical levels [3]. Thus, temperature-dependent oxygen limitation first lowers whole-animal aerobic scope (and hence performance), followed by the onset of anaerobic metabolism, finally resulting in heat damage to proteins, membranes and cells, and ultimately death [2], [3], [6], [7].

Although argued to hold for ectotherms in general [3], most of the evidence for oxygen limitation at thermal extremes to date comes from a variety of marine taxa, including fish, crustaceans, bivalves, and annelids [3], [ 5 and references therein]. Recent studies of terrestrial isopods and beetles [7], [8] question the generality of this mechanism, suggesting that upper and lower limits are decoupled in terrestrial arthropods, and showing no increase in critical thermal maximum (*CT*_max_) with hyperoxia. Additionally, whilst hypoxia decreased *CT*_max_ in isopods, an effect was only seen at extreme hypoxia in beetles, casting doubt on the degree to which oxygen limitation sets upper thermal limits in tracheates. From a functional perspective, both the fact that the tracheal system constitutes a single-step gas exchange system and their high efficiency of oxygen delivery, would indeed seem to preclude oxygen limitation as a major mechanism setting upper thermal limits in terrestrial insects [8]. In addition, terrestrial insects can maintain relatively constant internal oxygen levels, across steep clines in external supply, by adjusting ventilation rates or through compensatory developmental changes in tracheal length, diameter or branching [9], [10].

Not all insect species and life-stages may be equally sensitive to temperature-dependent oxygen limitation however and there remains a need for additional studies across the range of morphologies and lifestyles seen in extant insects [11]. In particular, many insects have aquatic larval stages, where the lower oxygen content and diffusion rates in water compared to air dramatically reduce available oxygen [12], [13], making oxygen limitation more likely. In addition, larger species or individuals may be more prone to temperature-dependent oxygen limitation because of longer diffusion pathways [4]. Similarly, if body size ultimately constrains an animal's capacity to supply its tissues with oxygen, this would explain why animals attain larger sizes in the cold, where metabolic rates are reduced [13], [14]. Such size based oxygen limitation is also central to the leading hypothesis regarding insect gigantism in the late Palaeozoic [15].

Here we provide the first formal test of oxygen limitation in a freshwater tracheate, the stonefly *Dinocras cephalotes* (Curtis, 1827). Their large, aquatic larvae rely predominantly on tegument respiration, making this an ideal species to study the principle of oxygen limitation. We examine the effects of hypoxia and hyperoxia on *CT*_max_ and test whether variation in *CT*_max_ amongst individual larvae is related to their oxygen consumption. Given the central role of oxygen in hypotheses on limits to both thermal tolerance and body size [6], [13], [15]–[17], we also discuss how the results of our study may improve our understanding of the role of oxygen in setting body size limits in arthropods. A second reason to discuss insect gigantism, stems from the fact that many insects exhibiting gigantism [18] apparently had aquatic larvae, although establishing larval lifestyle from fossil data can be difficult [19], [20]. We do not suggest that insect gigantism is restricted to insects with early aquatic life stages. The now extinct order of Palaeodictyoptera contained several species that displayed marked gigantism and available fossil evidence suggests a terrestrial larval stage [21], [22]. However, gigantic insects do seem to have been predominantly aquatic; the aquatic larval stage is held to be a common ancestral ground plan [20] for the giant griffenflies (Protodonata, sometimes also referred to as dragonflies) and giant mayflies (Ephemeroptera) of the late Palaeozoic, as well as the stoneflies (Plecoptera) arising in the Permian. Additionally the gigantic Carboniferous myriapods, the arthopleurids, are usually considered amphibiotic, where the early life stages are aquatic [20].

To date, attempts to understand insect gigantism in the late Palaeozoic have mainly been approached from the perspective of (fossilized) terrestrial adults. Yet, if oxygen limitation at thermal extremes operates differently for aquatic larvae and terrestrial adults, approaching the problem of historical gigantism from a larval perspective instead may similarly shed new light on the possible role of oxygen in setting insect body size limits. A larval view seems furthermore promising as insects with aquatic larval stages can circumvent the problem of structural support during their growing phase [15], while their flying adults overcome leg space limitations governing tracheal space limitations in walking insects [23].

Here we demonstrate that upper thermal limits in the aquatic life stages of an insect do indeed respond to external oxygen supply and that these are related to the oxygen consumption of individual larvae. These findings extend the generality of the hypothesis of oxygen limitation of thermal tolerance, suggest that oxygen constraints on body size may be stronger in aquatic environments, and that oxygen toxicity may have actively selected for gigantism in the aquatic stages of Carboniferous arthropods.

## Results

**Figure 1:**
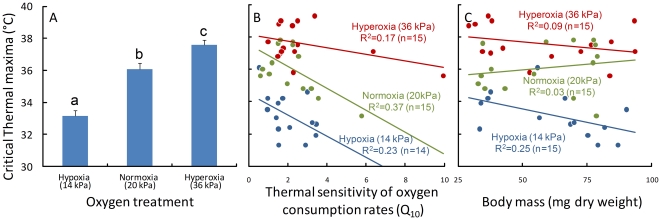
Differences in critical thermal maxima (*CT*_max_) in the stonefly *Dinocras cephalotes* at three different levels of oxygen (a), the relationship between *CT*_max_ of the stonefly nymphs and their thermal sensitivity in oxygen consumption (b) and their body mass (c). Differences in *CT*_max_ were consistent with the mechanism of oxygen limitation: hypoxia lowered *CT*_max_, while hyperoxia increased *CT*_max_ (a) and thermal maxima were lower for individuals which strongly increased their oxygen consumption rates at higher temperatures (high Q_10_ values). Each bar represents the average (± s.e.) of 15 nymphs. Letters indicate significant differences (*P<0.05*; Tukey HSD post hoc test following an anova including only oxygen treatment: F_2,41_ = 44·06, P<0·001).

Critical thermal maximum (*CT*_max_) differed significantly across oxygen treatments (Table 1) being raised by 1·53°C in hyperoxia and lowered by 2·92°C in hypoxia (Fig. 1A). Individual larvae differed in thermal sensitivity for oxygen consumption rates (expressed by Q_10_ values), which significantly affected their *CT*_max_ (Table 1). Thermal maxima were approximately 1°C lower for individuals with a high thermal sensitivity (Q_10_ value of 3) compared to individuals with a low thermal sensitivity (Q_10_ value of 1) (Fig. 1B). While we did not find a direct relationship between body mass and *CT*_max_ (Table 1; Fig. 1C), larger instars had higher Q_10_ values in comparison to smaller instars (*t*-test, *t*_1,41_ = 2·19, *P* = 0·038). This suggests that potential effects of body mass are mediated primarily through increased thermal sensitivity, something which seems most apparent at hypoxia (Fig. 1C).

**Table 1:** Statistical analysis of critical thermal maxima in relation to ambient oxygen levels, larval oxygen consumption and body mass.

Source	SS (Type III)	d.f.	*F* ratio	*P*-value
Oxygen treatment	131.897	2	45.338	**<0.0001**
Q_10_ oxygen consumption rates	12.132	1	8.341	**0.0062**
Body mass (mg dry weight)	.356	1	.245	0.6234

Ancova statistics on critical thermal maxima. Significant results are indicated in bold. Thermal maxima were highest for hyperoxia (36 kPa) and lowest for hypoxia (14 kPa). In addition thermal maxima were lowest for larvae which consumed more oxygen at higher temperatures. (SS = Sum of squares; d.f. = degrees of freedom).

## Discussion

Here we find strong evidence for the idea that a mismatch between external oxygen supply and internal oxygen demand can set thermal limits in aquatic insects [13]: hypoxia lowered thermal maxima, whilst hyperoxia increased them (Fig. 1A). At the same time, individuals that strongly increased oxygen consumption at elevated temperatures had lower thermal maxima (Fig. 1B). These results contrast with previous investigations on terrestrial adult insects, and provide a proof of principle that oxygen limitation can set upper thermal limits in aquatic insect larvae. Drawing on differences in ontogeny (closed trachea) and ecology (aquatic habitat), two explanations could be made for the apparent mismatch between oxygen supply and demand in the stonefly nymphs, causing the onset of oxygen limitation at thermal extremes.

First, oxygen delivery may be impeded in stonefly nymphs because of their closed tracheae and because of the lower oxygen content and diffusion rates in water compared to air. At higher temperatures, more oxygen is available to an aquatic organism because of the higher diffusivity of oxygen, yet scope for aerobic metabolism is nevertheless reduced as increases in organismal oxygen demand exceed increases in oxygen supply [13]. Although a closed tracheal system still represents a one-stage oxygen delivery system, oxygen delivery is more likely to become rate limiting at higher temperatures because of the additional step of oxygen diffusion across the epithelium. The absence of air sacs in larvae may further limit oxygen delivery rates by increasing the relative importance of diffusive rather than convective movement of oxygen in the trachea [9], although the compression and expansion of the trachea themselves [24] may in some cases generate substantial convective movement [25]. An increased difficulty of oxygen delivery in an aquatic environment fits with the fact that *CT*_max_ at normoxia is generally reported to be higher for terrestrial than aquatic arthropod life stages [7], [8], [26]–[29]. As *CT*_max_ is reached at lower temperatures in aquatic taxa, this is less likely to be a result of thermal damage at the cellular level such as the disruption of membrane structure and problems associated with protein folding [30], [31], which would make oxygen limitation more decisive in setting thermal limits in aquatic life stages, rather than one of several factors as suggested for terrestrial insects [7], [8]. One assumption here is that aquatic taxa have not considerably altered their membrane fluidity and protein activity in response to the more stable, often lower thermal regime in aquatic habitats (see below). If oxygen is more important at less extreme thermal maxima relative to other factors (membrane fluidity and protein stability), it would explain why *CT*_max_ increased with hyperoxia in the stonefly nymphs studied here (Fig. 1A) and in aquatic mayfly nymphs studied by Whitney [26], but not in the terrestrial tracheates with open tracheal systems studied so far [7], [8].

Second, changes in external oxygen levels may have stronger impacts on internal oxygen levels in aquatic invertebrates, and hence thermal tolerance, if their oxygen regulatory capacity is more limited than that of terrestrial invertebrates. Insects are forced to regulate internal oxygen levels within a fairly narrow range, balancing the risk of asphyxiation with that of oxygen toxicity [10], [32], [33]. Whereas terrestrial insects can simply open or close spiracles to regulate oxygen uptake, such regulation is unavailable to aquatic stages with closed tracheal systems such as *Dinocras*. Equally, fluctuations in ectotherm oxygen consumption are reduced in aquatic habitats owing to their thermally buffered nature. In short, the ability to regulate internal oxygen levels is inherently limited in taxa with closed tracheal systems, while the need to do so is lower in aquatic habitats. With poor regulation of oxygen intake in *Dinocras*, it is perhaps not too surprising to find a relationship between *CT*_max_ of individual stonefly nymphs and their thermal sensitivity in oxygen consumption (Fig. 1B). As these measurements of oxygen consumption were performed at ecologically realistic temperatures, aerobic scope for feeding, growth and reproduction may be likewise affected by the interplay between external oxygen supply and organismal oxygen demand, although the thermal limits associated with these performance measures will be lower than those for the short-term survival reported here [2], [4], [6].

In each of the above explanations the conditions that make oxygen limitation more likely arise from both the aquatic nature (lower oxygen availability and higher thermal buffering) and the closed tracheal system (limiting oxygen delivery and regulatory capacity). Consequently, oxygen limitation may be especially likely for insects that have life stages with closed tracheal systems and live in an aquatic or essentially aquatic environment (e.g. endoparasites, endophytic species, some rotten wood borers, rotten fruit specialists, etc.). Thus, many insects may be affected by oxygen limitation at some stage during their life cycle; indeed different life stages vary in their susceptibility to hypoxia [10], [34], [35] and thermal tolerance [36].

Similarly, both of the above explanations for the onset of oxygen limitation at thermal extremes could underlie insect gigantism. While they do not preclude additional evolutionary routes toward gigantism (which seems most probable for entirely terrestrial taxa like the Palaeodictyoptera), they could explain why gigantism was apparently frequent amongst arthropods with juvenile aquatic life stages. Importantly, each explanation makes very different predictions. The first, of impeded oxygen delivery, follows the existing explanation that increased atmospheric levels of oxygen in the late Palaeozoic permitted the evolution of larger body sizes. The basic difference is that oxygen limitation first sets in at the larval stage, either owing to the lower availability of oxygen in water compared to air [12], [13] or the additional barrier of diffusion across the epithelium.

Oxygen limitation in larvae fits with the observation that oxygen delivery does not seem to become much more challenging for larger bodied adults of terrestrial insects [10], [15]. Although larger animals are predicted to be more prone to oxygen limitation, such size dependency may only be evident under certain conditions [15], [17], given that larger individuals may have modified respiratory structures, and change their respiratory behaviour to compensate for reductions in oxygen supply capacity associated with larger size [10], [15]. Costs associated with such compensatory changes may include tracheal hypertrophy [9], [23], or increased thermal sensitivity (see results). These costs may be reflected in the long term, underlying observed variation in body size across environmental gradients of temperature or oxygen availability [13], [14]. Here we found support for size related performance in the hypoxic treatment only (Fig. 1C). A possible explanation for this is that the tracheal network of the larger individuals, which developed under normoxia in the field, was of insufficient capacity when larvae approached their thermal limits under hypoxia. At higher levels of oxygen, other limits may have set in that were less dependent on size, involving the failure of oxygen delivery systems operating at the cellular level [14].

A fundamental problem with a passive oxygen ceiling constraining maximum body size is that it is inferred from extant taxa having physiologies optimized to normoxic conditions and therefore may not reflect the evolutionary limit of tracheal breathing [37]. The second explanation of limited oxygen regulatory capacity recasts oxygen as an active driver of gigantism by focussing on the risks of having too much oxygen, rather than too little. In aquatic larvae with closed tracheal systems and limited ability to regulate internal oxygen levels, internal oxygen levels would be expected to closely track environmental oxygen levels. Whilst species are likely to have experienced and evolved responses to cope with periods of hypoxia [38], the same may not apply for hyperoxia, putting aquatic larvae at greater risk of oxygen poisoning than terrestrial adults [39]. If large body sizes are more sensitive to hypoxia and asphyxiation, they may equally confer protection from oxygen toxicity [40], constituting an antioxidant response [41].

In support of oxygen as an active driver of increasing body size, Loudon [9] found that beetle larvae increased in body mass when they were transferred from hypoxia to normoxia during their development. The logic is that larvae which started their development in hypoxia increased their tracheal size, but could not decrease them again upon returning to normoxia, as the new trachea are built around the old trachea of the previous larval instar. Although increasing body mass entails costs, body mass results from the net effect of many different factors [14]. During development, internal hypoxia acts as a signal and may stimulate tissue differentiation instead of growth, thus affecting the size reached. Similarly hyperoxia may trigger an increase in body size as a readily available way to effectively escape oxygen toxicity, possibly enabled through lower costs in ventilation or tracheal investments [10], [40], [42]. Hence hyperoxia may actively drive evolutionary increases in body mass, even in small insects [40]. Direct evidence for oxygen toxicity in a range of freshwater invertebrate species is provided by Fox & Taylor [32] who found that smaller juvenile stages are more sensitive to hyperoxic conditions than their larger aquatic adults.

An active selection for larger body sizes under hyperoxia would fit with the reappearance of giant mayflies in the putative high oxygen atmosphere at the end-Cretaceous [43] and the persistence of giant insects during putative lower levels of atmospheric oxygen. Examples of the latter include large griffenflies (Protodonata) in the late Permian [44] and abnormally large dragonflies (Odonata) during the Triassic/Jurassic [45]. Similarly, oxygen as an active driver of gigantism would predict a shift in size spectra such that average size increases, rather than a unilateral broadening of size spectra where only the body size of the largest species increases, as predicted by a passive oxygen ceiling. While establishing changes in modal sizes from the fossil record is fraught with difficulties, size spectra in extant amphipod assemblages would support oxygen as an active driver, where not only maximum body size within an assemblage, but also average and even minimum body size is greater at higher ratios of oxygen supply to demand [13], [46].

Thus, a larval view of Paleozoic hyperoxia-enabled gigantism in insects may be very informative. We suggest the aquatic larval stage as a route to gigantism, explaining the predominance of aquatic life stages amongst extinct gigantic insects. In aquatic juvenile stages, impeded oxygen delivery may have resulted in stronger constraints on body size, whilst larger body sizes may have been actively selected for to avert oxygen toxicity. The widespread gigantism in marine species further supports this hypothesis, with examples of gigantism [47], [48] found predominantly at times when oxygen levels were perhaps not higher than current ones, but rising [49], effectively putting the animals at risk of oxygen poisoning. Each of the two explanations for a larger role of oxygen in aquatic stages yields testable predictions concerning the relative rates of evolution of larger and smaller body sizes, and the shifting or broadening size spectra.

## Materials and Methods

Animals were collected from the River Dart, Devon, UK and maintained in the lab at 10±1°C in a 12 L∶12 D regime. They were kept in a flow-through aquarium (10 L·min^−1^), fed with artificial pond water [50], buffered and diluted to reflect the pH and conductivity of the field site (pH 6.4–6.6, 70–150 µS·cm^−1^) and fed chironomid larvae. Oxygen consumption was measured for each larva at 10°C and 15°C using closed glass respiration chambers of 67.5–68.5 ml. The chambers were immersed in a temperature controlled bath (±0.1°C) and stirred using underwater magnetic stirrers to ensure mixing of water. Respiration chambers were fitted with a fine nylon mesh forming a false bottom to prevent contact between the larvae and the magnetic stirrer bar. Individuals were allowed to acclimate for 10 min before the chambers were closed and left for 60 min. Oxygen content was measured before and after using an O_2_ electrode (1302 Oxygen Electrode, Strathkelvin Instruments) that was connected to a calibrated meter (Oxygen Meter 929, Strathkelvin Instruments). On average, larvae depleted oxygen levels only by 5% (with a maximum of 18%) and oxygen consumption was expressed as µg O_2_ (mg wet mass)^−1^ h^−1^. Oxygen consumption of larvae at 10°C was significantly correlated with their consumption at 15°C (Partial correlation, corrected for differences in body mass: r = 0.511, *P*<0.001), justifying the calculation of Q_10_ values for individuals. One individual was removed from further analysis as abnormally low oxygen consumption measured at 10°C resulted in an unrealistic Q_10_ value of 99.The Q_10_ values calculated for each specimen were correlated with the *CT*_max_ values of the exact same individuals.

To assess *CT*_max_ at normoxia, animals were placed in small flow-through chambers (70×70×30 mm; flowrate 0.016 L·s^−1^) fed with water from a 25 L header tank passing through a tubular counter current heat exchanger. Water in the header tank was of the same composition as that used to maintain animals, and was bubbled with a mixture of 20% oxygen and 80% nitrogen, obtained using a gas-mixing pump (Wösthoff, Bochum, Germany), and covered with 18 mm thick expanded polystyrene sheeting to prevent equilibration with the atmosphere. Before the start of the experiment, animals were left to acclimate for 1 h at the starting temperature of 10°C, after which temperature in the experimental chambers was increased at 0.25°C min^−1^, using a Grant R5 water bath with a GP200 pump unit (Grant Instrument (Cambridge) Ltd, UK), connected to the heat exchanger. Temperatures were logged using a HH806AU digital thermometer (Omega Engineering Inc., USA). *CT*_max_ was recorded as the point at which animals no longer showed any body movement or muscular spasms. Animals which were at this point transferred to fully oxygenated water of 10°C recovered with no apparent lasting damage. Below critical maxima, larvae initiated in repeated swimming behaviour (around 29°C; interpreted as attempts to escape experimental conditions) and fell upon their backs (around 30°C) and an onset of spasms was observed (around 31°C). We used a dynamic method (acute exposure to changing temperatures) in contrast to most work performed on marine taxa, which used static methods (chronic exposure to constant temperatures [3], ). As faster rates of warming can result in higher critical thermal maxima [51], [52], we employed the same rate of warming as employed in previous studies on terrestrial insects to minimize confounding effects arising from methodological differences in a direct comparison of our results with theirs. To assess *CT*_max_ at hypoxia (5% O_2_, 95% N_2_) and hyperoxia (60% O_2_, 40% N_2_), the gas mixture was adjusted 10 min after placing the animals in the small flow-through chambers. In this way animals were gradually exposed to hypoxic and hyperoxic conditions during acclimation. In several test runs, oxygen levels were measured after adjusting the gas mixture to determine when and at which value oxygen levels stabilized. From these measurements the relative contributions of the gas mixture and normal air to the (hypoxic or hyperoxic) test water could be determined. The oxygen levels stabilised after 1 hour at 36 kPa (34–38) and 14 kPa (13.3–14.8) for the hyperoxia and hypoxia treatment, respectively.
